# Self Esteem and Organizational Commitment Among Health Information Management Staff in Tertiary Care Hospitals in Tehran

**DOI:** 10.5539/gjhs.v7n2p328

**Published:** 2014-12-12

**Authors:** Farahnaz Sadoughi, Kamal Ebrahimi

**Affiliations:** 1 School of Health Management and Information Science, Iran University of Medical Science, Tehran, Iran; 2 School of Health Management and Information Science, Iran university of medical Science, Tehran, Iran

**Keywords:** self esteem, organizational commitment, affective commitment, continuance commitment, normative commitment

## Abstract

**Background:**

Self esteem (SE) and organizational commitment (OC) have significant impact on the quality of work life.

**Aim:**

This study aims to gain a better understanding of the relationships between SE and OC among health information management staff in tertiary care hospitals in Tehran (Iran).

**Methods:**

This was a descriptive correlational and cross sectional study conducted on the health information management staff of tertiary care hospitals in Tehran, Iran. A total of 155 participants were randomly selected from 400 staff. Data were collected by two standard questionnaires. The SE and OC was measured using Eysenck SE scale and Meyer and Allen’s three component model, respectively. The collected data were analyzed with the SPSS (version 16) using statistical tests of of independent T-test, Pearson Correlation coefficient, one way ANOVA and F tests.

**Results:**

The OC and SE of the employees’ were 67.8, out of 120 (weak and 21.0 out of 30 (moderate), respectively. The values for affective commitment, normative commitment, and continuance commitment were respectively 21.3 out of 40 (moderate), 23.9 out of 40 (moderate), and 22.7 out of 40 (moderate). The Pearson correlation coefficient test showed a significant OC and SE was statistically significant (P<0.05). The one way ANOVA test (P<0.05) did not show any significant difference between educational degree and work experience with SE and OC.

**Conclusion:**

This research showed that SE and OC are moderate. SE and OC have strong correlation with turnover, critical thinking, job satisfaction, and individual and organizational improvement. Therefore, applying appropriate human resource policies is crucial to reinforce these measures.

## 1. Introduction

Previous studies have indicated the main factors influencing the productivity of organizations at macro or micro levels, where the human resources plays a crucial role in these factors (Ahmad & Schroeder, 2003; Mathauer, & Imhoff, 2006; Dorgham, 2012). The proper management of human resources depends on understanding their attitude towards themselves and organization (Dussault & Dubois, 2003). To achieve this goal, investigating the self esteem (SE) and organizational commitment (OC) is one of the most important approaches (Pierce & Gardner, 2004). SE and OC as complementary to each other, focus individual and social aspects, respectively.

SE is one of the most challenging issues in the psychology, considered as behavior predictor, strong relief for anxiety and substantial solution to organizational problems (Lee & Mitchell, 1994; Van Dyne et al., 2000; Elloy, & Patil, 2012). SE can maintain the feeling of happiness and competence of human being when facing the life’s challenges (Pierce & Gardner, 2004) Occupational success leads to high SE and people with high SE better than their peers with low SE in groups (Baumeister, Campbell, Krueger, & Vohs, 2003).

OC is more related to social interactions of people so that they reveal it in the work environment according to their preferences, talents, attitudes and cognitions. In addition, it plays a promising role in the success of organization (Judge & Bono, 2001). OC has significant effect on the job abandonment and organizational challenges, making it an important factor in similar studies (Allen, Weeks, & Moffitt, 2005; Stallworth, 2003). There are various definitions for OC. Among them Meyer and Allen definition is more famous and acceptable among others (Meyer & Allen, 1991; Lee et al., 2001). Meyer and Allen state that an employee’s commitment reflectes a desire, need and obligation to maintain membership in an organization. They propose three distinct aspects of an employee’s OC: affective commitment, normative commitment and continuance commitment (Meyer & Allen, 1991).

OC has received considerable interest from organizational researchers since its inception. Therefore, it has been tested in a variety of research fields such as: job satisfaction (Williams & Anderson, 1991; Clugston, 2000; Fu, & Deshpande, 2013; Ibrahem, Elhoseeny, & Mahmoud, 2013; Tnay et al., 2013), learning organization (Pool & Pool, 2007) and turn over (Matz et al., 2013; Griffin et al., 2010). In this regard, some studies have assessed the relation between SE and variables such as critical thinking (Suliman & Halabi, 2007) mental health (Baumeister et al., 2003) and job satisfaction (Judge & Bono, 2001).

Therefore, SE and OC representing individual and social aspects can detect the efficiency rate of employees. Previous studies on Iranian hospitals have demonstrated different occupational problems including occupational stress, staff shortage, lack of promotion, and turnover (Manafi, 2012; Aghdasi, Kiamanesh, & Ebrahim, 2011; Mosadeghrad, Ferlie, & Rosenberg, 2011).

Human resources in health information technology have important role in quality, efficiency, and reducing medical errors (Center, 2010; Menachemi, & Collum, 2011; Blumenthal, 2011). Ministry of Health and Medical Education in Iran has a national level program for developing information systems in hospitals which is profoundly related to human resources of health information technology (Farzadfar, 2014; Tavakoli et al., 2013). Furthermore, OC and SE scales are useful in finding the attitude of these staff in individual and organizational aspects and improving these aspects.

The present study aims to investigate the relationships between SE and OC among the health information technology staff of the tertiary care hospitals in Tehran (Iran) to gain a better understanding of the relationships between SE and OC.

## 2. Methods

This study was a descriptive correlational and cross sectional survey conducted on 400 employees of health information technology department of the tertiary care hospitals of Tehran in 2010. The sample size of 155 was determined by Morgan Table and 155 155 participate in research. Eighteen hospitals were selected through a random sampling method. One hundred and fifty five questionnaires were used for all participants. The SE and OC was measured using Eysenck SE scale and Meyer and Allen’s three component model, respectively. These questionnaires are standard. Validity and reliability of SE questionnaire had been verified by previous studies (Khayatmoghadam, 2008; Haghirosadat, 2010). In addition, the OC questionnaire had been verified by previous studies (Hahirosadat, 2010; Bergman, 2006; Cheng, 2003; Meyer, & Allen, 1991). The collected data were analyzed with the SPSS (version 16) using statistical tests of independent T-test, Pearson Correlation coefficient, one way ANOVA and F tests.

## 3. Results

The staff involved in the study had the mean age below 35 years old (75%). The majority of them (75%) had a bachelor’s degree in Health Information Technology major.

Total score for SE and OC measured by Eysenck and Meyer and Allen scale is 30 and 120, respectively.

Total score for affective, continuance and normative commitment is 40. Employees’ scores are showed in Table 1.

**Table 1:** The mean scores of SE, affective commitment, normative commitment, continuance commitment and OC related to the employees of health information technology department in the tertiary hospitals in Tehran

	N	Minimum	Maximum	Mean(Std. Deviation)
SE	155	9.5	27.0	21.±4.2
Affective Commitment	155	10	40	21.3±5.7
Continuance Commitment	155	12	33	22.7±3.5
Normative Commitment.	155	11	36	23.9±3.7
OC(Total)	155	36	102	67.8±9.9

Findings show that the employees’ OC and SE were 67.8, out of 120 (weak) and 21.0 out of 30 (moderate), respectively. The values for affective commitment, normative commitment, and continuance commitment were respectively 21.3 out of 40 (moderate), 23.9 out of 40 (moderate), and 22.7 out of 40 (moderate) (Table 1).

The independent T-test (p<0.05) was conducted to compare the differences the marital status and OC (t=.045 df=150 sig.964), continuance commitment (t=.927 df=150 sig.355) and SE (t=1.188 df=150 sig.237). The Pearson correlation coefficient (r) test showed no statistically significant relationship between affective, normative and continuance commitment with SE. However, the relationship between OC and SE was statically significant (p<0.05). The detailed results of the statistical tests are presented in Table 2.

**Table 2:** Pearson’s correlation coefficient (r) among affective, normative, continuance, OC and SE of the employees of health information technology department in the tertiary hospitals in Tehran

		Affective Commitment	Normative Commitment.	Continuance Commitment	OC (Total)
SE	Pearson Correlation	-.151	-.139	- 0.05	-.155
	Sig.	.06	.08	.532	0.05

The ANOVA statistical test show significant effect of age on OC (F =1.934; df=33; sig.0.005), whereas no significant effect of age was found on SE (F =1.148; df=33; sig.0.289) (Table 3).

**Table 3:** The differences of average OC among different age groups of employees of the health information technology department in the tertiary hospitals in Tehran

		Sum of Squares	df	Mean Square	F	Sig.
OC	Between Groups	527.829	33	159.72	1.93	.005
	Within Groups	9991.468	121	82.57		
	Total	15262.297	154			

The findings show no significant difference between age and affective, normative and continuance commitment and SE. Also statistical test (P<0.05) did not show any significant difference between educational degree and work experience with SE, affective commitment, normative commitment, continuance commitment and OC.

## 4. Discussion

The present study evaluated the relationships between SE and OC among health information management staff in tertiary care hospitals in Tehran (Iran). The average scores of SE were frequently moderate (22.3 out of 30) Haghirosadat reported this score as 21.8 (Haghirosadat, 2010). In addition, our findings show the mean score of OC as modest that are in agreement with the findings of Haghirosadat (Haghirosadat, 2010) and (Khayatmoghadam, 2008). Our results did not indicate any significant relationship between SE and OC, whereas Haghirosadat (Haghirosadat, 2010) and Khayatmoghadam (Khayatmoghadam, 2008) showed significant relationship between the two variables. The significance level of the present study was close to 0 0.05. There was correlation between SE and continuance commitment which supports the results of Khayatmoghadam (Khayatmoghadam, 2008), Contray, the findings of Haghirosadat there is a significant relation between SE and affective commitment in the studies of Khayatmoghadam (Khayatmoghadam, 2008) and Haghirosadat (Haghirosadat, 2010). However, this significance was not reported in this study. Among other similar studies, only Khayatmoghadam (2008) reported a significant relation between SE and normative commitment (Khayatmoghadam, 2008).

In this study, normative commitment of employees acquired higher score than continuance and affective commitment and mean score of OC was 67 from 120. The OC is a psychological status in the three component model of Meyer and Allen that includes: desire, need and obligation named affective commitment, continuance commitment and normative commitment, respectively. Meyer and Allen mapped affective and normative commitments with attitudinal dimension and continuance commitment with behavioral dimension (Meyer & Allen 1991). Affective commitment is defined as the employee’s positive emotional attachment to the organization. When an employee is loyal to a particular organization and so remains in there, continuance commitment occurs. The results are complex. Some studies showed continuance commitment is one dimensional, whereas other studies indicated as two dimensional commitments including costs to leaving organization and a lack of alternative employment opportunities (Allen et al., 2001). In current study, there was no significant difference among work experience, OC and SE. Van dyne reports that more work experience leads to an increase in the organizational commitment, because people during the job years are more adapted with their colleagues and organization and usually find better work opportunities in the organization (Van Dyne et al. 2000). Lee concluded that if employees had no experience of the successful situations, work experience would have an inverse relation with OC (Lee et al., 2001).

SE is a growing field (Brown, 2014; Orth, Robins, & Widaman, 2012). New research in this particular context focuses on internet, new media usages and social networking sites (Ehrenberg et. al., 2008; Steinfield et al., 2008; Kramer & Winter, 2008; King, & Delfabbro, 2014).

## 5. Conclusion

The findings of this study showed that SE and OC of the employees of health information technology department in the tertiary hospitals in Tehran are moderate. SE is a growing field and a very important feature among other features related to mental health. In other words, it is one of the most fundamental factors affecting mental health. In addition, SE is effective in the success and compatibility of people. The social aspect of SE is the OC that a person shows it in the workplace based on their preferences, talents, attitudes and cognitions. The OC has a key role in the employee’s loyalty and is the most important related factor defining performance, values and attitudes of the employees. Especially it is a kind of belief and internal feelings of a person affecting performance, adherence and judgment of him/her to the organization. In general, SE and OC affect the organization performance through improvement of job quality, job abandonment, job satisfaction, job stress, human resources output, critical thinking, stimulus for progress, education success rate, stress reducing and job conflict. Because of the importance of health information technology department, managers and policy makers of health systems should fulfill a proper planning to improve human resources management. In this regard, insufficient attention can lead to low rates of return on investment.

Our findings recommend further studies on attitudinal level, job satisfaction relations, SE and OC, organizational health and organizational justice of the employees of health information technology department.
